# The applicability and effectiveness of the cognitive behavioral therapy for insomnia (Smart CBT-I plus) online program in patients with insomnia disorder combined with anxiety and depression: a randomized controlled trial protocol

**DOI:** 10.3389/fpsyt.2025.1450275

**Published:** 2025-03-28

**Authors:** Yating Zhao, Fangmei Ge, Xin Luo, Jingru Li, Jing Zhang, Yi Ju, Jie Zhang, Yong Wang, Dongbin Lyu, Yiren Qiu, Chengmei Yuan

**Affiliations:** ^1^ Shanghai Mental Health Center, Shanghai Jiao Tong University School of Medicine, Shanghai, China; ^2^ Shanghai Putuo Mental Health Center, Shanghai, China

**Keywords:** online self-help intervention, insomnia comorbidities anxiety and/or depression, randomized controlled trial, cognitive behavioral treatment of insomnia, protocol, effectiveness and applicability

## Abstract

**Background:**

Insomnia is often accompanied by depression and anxiety, which can seriously affect people’s quality of life. Cognitive behavioral therapy for insomnia (CBT-I) is the first-line treatment, but the existing CBT-I ignores the intervention for anxiety-depressive symptoms, and has poor efficacy due to the lack of artificial support, poor compliance, the inability to spread widely and high dropping rate. A balance is needed between the convenience and efficiency of web-based technology and patient needs. Again in this context, an online WeChat applet (Smart CBT-I plus) will be developed with CBT-I technology as the core, integrating cognitive behavioral intervention modules for depression and anxiety.

**Objectives:**

This study will validate the effectiveness and applicability of Smart CBT-I plus by examining whether the Smart CBT-I plus study group will significantly reduce the distress of people suffering from insomnia with anxiety and/or depression symptoms compared to the psychoeducational group.

**Methods:**

In this parallel-group, randomized controlled trial, 180 patients seeking help for insomnia combined with anxiety and/or depression will be recruited, and they will be randomized with 60 patients being assigned to the psychoeducation group (control group), and 120 patients being assigned to the Smart CBT-I plus group (study group). Measurements will be taken at baseline, post-intervention, 6 and 12 month follow-up, at the same time, semi-structured qualitative interviews about the experience of using Smart CBT-I plus will be conducted with randomly selected patients from the study group.

**Results:**

The results will involve insomnia, depression and anxiety to explore its effectiveness, in-treatment dropout rates and subjective patient feedback to explore the applicability of Smart CBT-I plus.

**Future recommendations:**

Self-help platforms need to be more individually designed to reach a wider audience. Research aimed at a wider audience, such as the general public, will make the research more universal and the platform more meaningful.

## 1 Introduction

### 1.1 Background

Insomnia is a serious public health problem, 19% to 50% of adults report experiencing insomnia, and approximately 10% meeting the diagnostic criteria for insomnia disorders (1, 2). Insomnia seriously affects the quality and efficiency of life and is one of the causes of many psychiatric disorders, such as anxiety and depression (3, 4).

For a long time, medication has dominated the treatment of insomnia but they are difficult to cure insomnia and have many side effects (5–7).Cognitive Behavioral Treatment of Insomnia (CBT-I) is a psychotherapy that addresses maladaptive behaviors and the dysfunctional concepts that perpetuate sleep problems, and is effective in improving sleep when treated in person by a trained therapist (8, 9). Studies have shown that the short-term effects of CBT-I are comparable to medication (9), while long-term efficacy is superior to medication (10). Several meta-analyses in recent years have shown that CBT-I is effective in different types of insomnia, including primary insomnia versus co-morbid insomnia (11), middle-aged and older adults (12, 13) and children and adolescents (14, 15).

In an early study investigating the relationship between insomnia and mental illness, 40% of them suffered from psychiatric disorder, most commonly anxiety (24%) or depression (14%) (3, 16, 17). Insomnia is closely related to anxiety and/or depression (18–20), and they have a mutually sustaining relationship (20–22). This suggests that improving concomitant anxiety and depressive symptoms in insomniac subjects is clinically important (23).

CBT-I has been gradually and widely used in the treatment of insomnia, but it has strong limitations, which are mainly reflected in the poor durability and stability of the efficacy, and the high relapse rate (24–26). Offline face-to-face CBT-I focuses on the treatment of “insomnia” itself, but neglecting the impact of negative emotions such as anxiety and depression on sleep. As offline face-to-face CBT-I is difficult to be widely promoted in practice due to problems such as expensive, time-consuming and lack of professional therapists (27), online or non-online self-help CBT-I, which helps patients achieve self-help treatments with the help of carriers such as books, computers, and mini-palm devices, has gradually appeared and has been proved to have a certain degree of efficacy (20, 28). However, they can also be undermined by a lack of professional guidance and the patient’s difficulty in accurately understanding the key points of the treatment.

Online self-help CBT-I programs relying on the Internet make it easier for users to receive different forms of information at the same time, have a higher degree of interactivity than non-online self-help CBT-I programs, and are able to provide more timely feedback (20). In contrast, online self-help CBT-I treatments in which some support is given to the patient through email, phone calls, etc., have a significant impact on its efficacy, which may help to increase adherence to the treatment, reduce the rate of disengagement, and thus promote efficacy (29). Therefore, online self-help CBT-I programs with a certain degree of interactivity and therapist support will have greater application value.

In this study, we developed a CBT-I based online intensive cognitive behavioral therapy for insomnia combined with anxiety and depression applet for patients with insomnia combined with anxiety and depression, which is a personalized online self-help CBT-I program for people with insomnia combined with anxiety and depressive symptoms. As mentioned above, because it is of great significance to improve the anxiety and depression symptoms associated with insomnia patients, the program developed in this study not only arranges the content of CBT-I, but also specially adds the treatment module for anxiety and depression, it’s called Smart CBT-I plus. Because Smart CBT-I plus intervention needs to be completed in a continuous 35 days, and the addition of the anxiety and depression module of the intervention is also a strengthening of the efficacy, it is therefore an intensive intervention. After the daily interventions targeting sleep disorders are completed, the program automatically reminds the patient to use the intervention modules for anxiety and depression. I call it Online Intensive Cognitive Behavioral Therapy for Insomnia (Smart CBT-I plus). Smart CBT-I plus is an online WeChat applet with human-computer interaction functions. When patients have doubts about the treatment content or need human intervention, they can go to the therapist’s mailbox on the homepage to give feedback, and the therapist will tailor the treatment to the patient’s needs and provide the necessary human support. CBT-I This study attempts to evaluate the effectiveness and applicability of Smart CBT-I plus.

### 1.2 Objectives

This study will conduct a prospective, parallel-group, randomized controlled clinical trial to investigate the effectiveness of Smart CBT-I plus in improving symptoms in patients with insomnia with anxiety and depression and the applicability, strengths and weaknesses, and directions for refinement of the procedure itself.

## 2 Methods

### 2.1 Trial design

This will be a single-center, parallel group, randomized controlled trial. A total of 180 patients with insomnia disorder combined with anxiety and/or depressive symptoms will be recruited and randomly assigned in a ratio of 1:2 to the online psychoeducation group (control group) and the Smart CBT-I plus treatment group (study group), and followed up at 6 months, and 12 months after the treatment. Both the control and study groups will be done online.

### 2.2 Setting and recruitment

**Figure 1:**
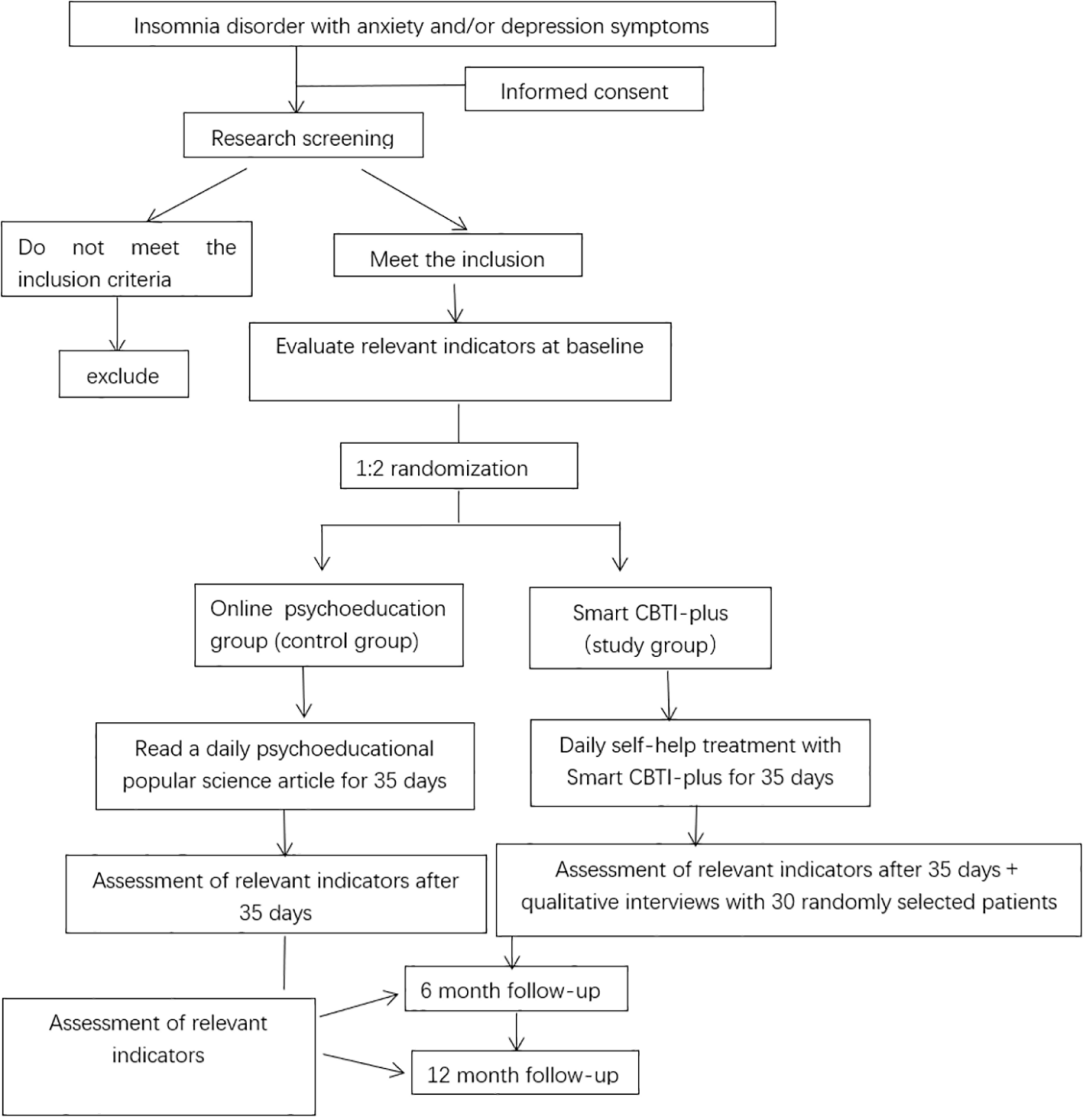
Study design and measurement time points.

We will enroll patients with insomnia with anxiety and/or depression symptoms in outpatient and inpatient clinics of Shanghai Mental Health Centre through poster publicity, online recruitment, and doctor recommendation. Patients who meet DSM-5 diagnostic criteria for insomnia and have comorbid psychological disorders will be recruited and referred by psychiatrists and psychotherapists with medical backgrounds at the Shanghai Mental Health Center, and then assessed by trained assessors.Patients who meet the inclusion criteria and sign the electronic informed consent forms (ICFs) will be randomly assigned to the control group and the study group, and the randomization will be confidential to the assessors. Next, a systematic semi-structured sleep assessment interview will be conducted by a professional psychotherapist who has undergone uniform training to assess the patient’s insomnia severity, social functioning, and basic upbringing history, to complete the conceptualization of the case of insomnia, to establish a preliminary therapeutic relationship, and to provide an introduction to the upcoming online intervention, which will last for approximately 1 hour. During this process, if the subject does not feel comfortable or has difficulty cooperating with the treatment, or if the therapist believes that the treatment program is not suitable for the subject, then the therapist will not enroll him/her in the group. The Insomnia Severity Index (ISI), Hamilton Depression Scale (HAMD) and Hamilton Anxiety Scale (HAMA) will be used for screening. Other criteria are listed in the “participants and eligibility” section. Participants who meet the criteria will be able to proceed to the next stage. The specific flowchart is found in **Figure 1**.

### 2.3 Participants and eligibility

Participants will be included in this study if(1)Considering that minors under the age of 18 do not have a strong sense of rights and interests to participate in the study, and elderly people over 65 have difficulty in using electronic devices, the inclusion criteria for this study is 18-65 years old age; (2)Currently meets DSM-5 diagnostic criteria for insomnia disorder; (3)Insomnia Severity Index(ISI)total score ≥15; (4)Concomitant anxiety and/or depressive symptoms, 14 points ≤ HAMA ≤ 29, 14 points ≤ HAMD-17 ≤ 23; (5) Adequate literacy and understanding to complete the required inspections and assessments for this study;(6)No sedative, antidepressant, anxiolytic, or antipsychotic medication was taken 2 weeks prior to enrolment, or the type and dose of the medication was stable for 4 weeks prior to enrolment. The type and dose of the drug is stable; (7)they volunteered to participate and signed the ICFs after fully understanding all aspects of the trial.

Participants will be excluded from the study if they meet the following criteria.(1)Other sleep disorders that meet DSM-5 diagnostic criteria, such as apnea syndrome, restless legs syndrome, etc.; (2) female who are pregnant, breastfeeding, or planning to become pregnant during the study period; (3) insomnia problems caused by alcohol or substance abuse; (4) severe cognitive problems; (5) patients with a previous or current diagnosis of bipolar and related disorders, obsessive-compulsive and related disorders, schizophrenia spectrum and other psychotic disorders, trauma and stress-related disorders, dissociative disorders, eating disorders, etc.; (6) patients with significant negative suicidal risk and a HAMD-17 item 2 score of ≥2; (7) patients with a history of epilepsy or other serious physical illnesses; (8) those who have been treated with MECT in the last month; (9) those who have received systematic psychological treatment for more than 3 consecutive months but found it to be ineffective; (10) those who are unable to tolerate the study setting; and (11) those who, in the opinion of the investigator, are unsuitable for the other conditions to participate in this study. For example, the researcher felt that the patient could not guarantee full and complete participation in the study, or that the patient would have a life change such as pregnancy plan that would not be suitable for inclusion in the study.

### 2.4 Randomization

Randomization will be based on a computer-generated list of random numbers randomly assigned in a 1:2 ratio to the online psychoeducational group (control group) and the online Smart CBT-I plus group (study group). Later, the computer program will use the generated random list to allocate patients, and the research assistant will inform the trained psychotherapist of the results of the randomized group of patients after allocation, and after the therapist’s interviews, allocate them to the corresponding control and intervention group fellows.

### 2.5 Intervention

#### 2.5.1 Medication

The patient’s original use of sleep aids, antidepressants, antipsychotics, etc. needs to be maintained at the same dose from the screening period and throughout the intervention period. In case of more pronounced insomnia problems, short-term use of zolpidem or zopiclone or dexzopiclone to assist sleep is permitted, but should not be used for more than 3 consecutive days and cumulatively for more than 2 weeks. Newly prescribed benzodiazepines, antidepressants, anxiolytics, antipsychotics, herbal preparations with sedative effects, and central stimulants are not allowed; self-administration of melatonin and melatonin-related products is not allowed.

#### 2.5.2 Online psychoeducational control group

The control group will receive one psychoeducation-related popular science article per day.

Psychological education about sleep disorders aims to provide more scientific and reasonable explanations for different types of insomnia, help patients correctly understand the symptoms of insomnia and the use of insomnia-related medications; teach patients how to cope with ambivalent insomnia, how to cultivate good sleep habits and daily routine; the sleep disturbances that may be encountered in different age groups and the measures to cope with them; sleep-related somatic reactions and common life events that cause sleep disorders. The relationship between insomnia and emotional problems such as anxiety and depression; somatic reactions related to sleep and how to cope with sleep disorders caused by common life events.

The researchers will send a psychological education article through wechat every day, and ask participants to reply “learned.”

Receiving relevant psychoeducation can promote symptomatic improvement (30), and at the same time, there is no immediate risk or indication for medical treatment, so the control group setting is ethically acceptable (31).

#### 2.5.3 Online Smart CBT-I plus group (intervention group)

##### 2.5.3.1 Technical overview and customization

After random assignment, patients in the Smart CBT-I plus group will enter the 35-day intervention. The Smart CBT-I plus will push fixed tasks for patients to complete every day for 35 days, and after completing the fixed tasks every day, patients can go to the homepage to review the relevant content on their own. If there is any doubt about the content of the treatment or if human support is needed, patients can go to the therapist’s mailbox on the homepage to give feedback, and the therapist will reply within 24 hours to answer questions. The intervention content of Smart CBT-I plus includes sleep hygiene education, relaxation training, sleep restriction, stimulus control and sleep cognitive correction. In addition to the insomnia treatment module, modules for depression and anxiety have been added. Smart CBT-I plus is a self-service psychological intervention platform on WeChat applets for patients with insomnia and emotional problems. The goal of this platform is to solve the current problem of limited psychotherapy resources, rather than replace psychotherapy. In order to design the knowledge structure of Smart CBT-I plus as well as to determine the content, four psychiatrists, three professional psychotherapists and three researchers at SMHC read extensively and studied in depth the literature and books related to sleep disorders, CBT-I, anxiety and depression. During the study design and platform development, several meetings were held to discuss the outline, write and revise scripts, create animations, and collaborate with a professional technology company to develop the program. After development, professionals and real patient groups were invited to do user experience and function tests, and user interviews were conducted for further modification and improvement. Eventually, the WeChat applet platform was completed in 1.5 years and is continuously improved and upgraded.

The “Smart CBT-I plus” applet platform is easily accessible on WeChat, China’s most frequently used application. Participants can easily use the platform from anywhere, with greater flexibility and anonymity.

It features CBT-I technology as the core, integrates cognitive behavioral interventions for anxiety and depression, and has human-computer interaction. It can reduce the number of trips that have to be made to the hospital, making it extremely convenient for patients. This saves travelling time and streamlines the treatment process. In addition, the platform’s online application reduces the amount of time spent in contact with clinicians while minimizing costs, which can make intervention treatment accessible to more populations.

By using the applet’s backend management system, researchers will be able to monitor and track all patients. When patients in either group are at risk of self-injury, suicide, or worsening symptoms of depression and/or anxiety, the researchers, in discussion with the study team, will have the option of stopping the study and assisting the patient to seek medical attention for emergency treatment. All of this will be done with informed consent at the start of the study.

##### 2.5.3.2 Integrative support approach

After logging into the WeChat app and completing the registration, the patient receives a short welcome message and a brief overview of the intervention’s format, modules, method of operation, and principles of treatment. At the same time, the patient is required to provide the necessary personal information, and after completing the opening, the patient will enter the daily intervention training.

Smart CBT-I plus is primarily based on the core techniques and principles of CBT-I, while incorporating Cognitive Behavioral Therapy (CBT) for anxiety and depressive symptoms. Stimulus Control Therapy, Sleep Restriction Therapy, Cognitive Therapy, Sleep Hygiene Education and Relaxation Training (20, 32), the 5 components were among the most commonly used components of CBT-I (33–35).

Patients with sleep disorders commonly co-morbidly suffer from anxiety and/or depression, which are closely related and interact with each other. Cognitive Behavioral Therapy (CBT) is a common treatment for symptoms of anxiety and depression with proven results (36, 37).

By the development of good sleep habits, the correction of sleep cognition, and the customization and implementation of personalized treatment strategies, sleep-related maladies and cognitions can be effectively reduced, and the development of chronic insomnia can be avoided through correct behavioral modification. The treatment consists of animation, audio, video, quizzes and email support from the therapist, making it a lively and varied program. The daily treatment and learning tasks are pushed to the patient between 3-5 minutes in length. The treatment tasks are linear, starting with a review of the previous day’s content, followed by the presentation of the day’s tasks, the last task of the day is a relaxation exercise, which can be done at any time throughout the day and with the audio of your choosing, followed by a reminder to go to the home page, which reminds you to go to self-help interventions for anxiety and depression, and then finally to the home page, which allows you to review the past treatments and learning content.

**Figure 2:**
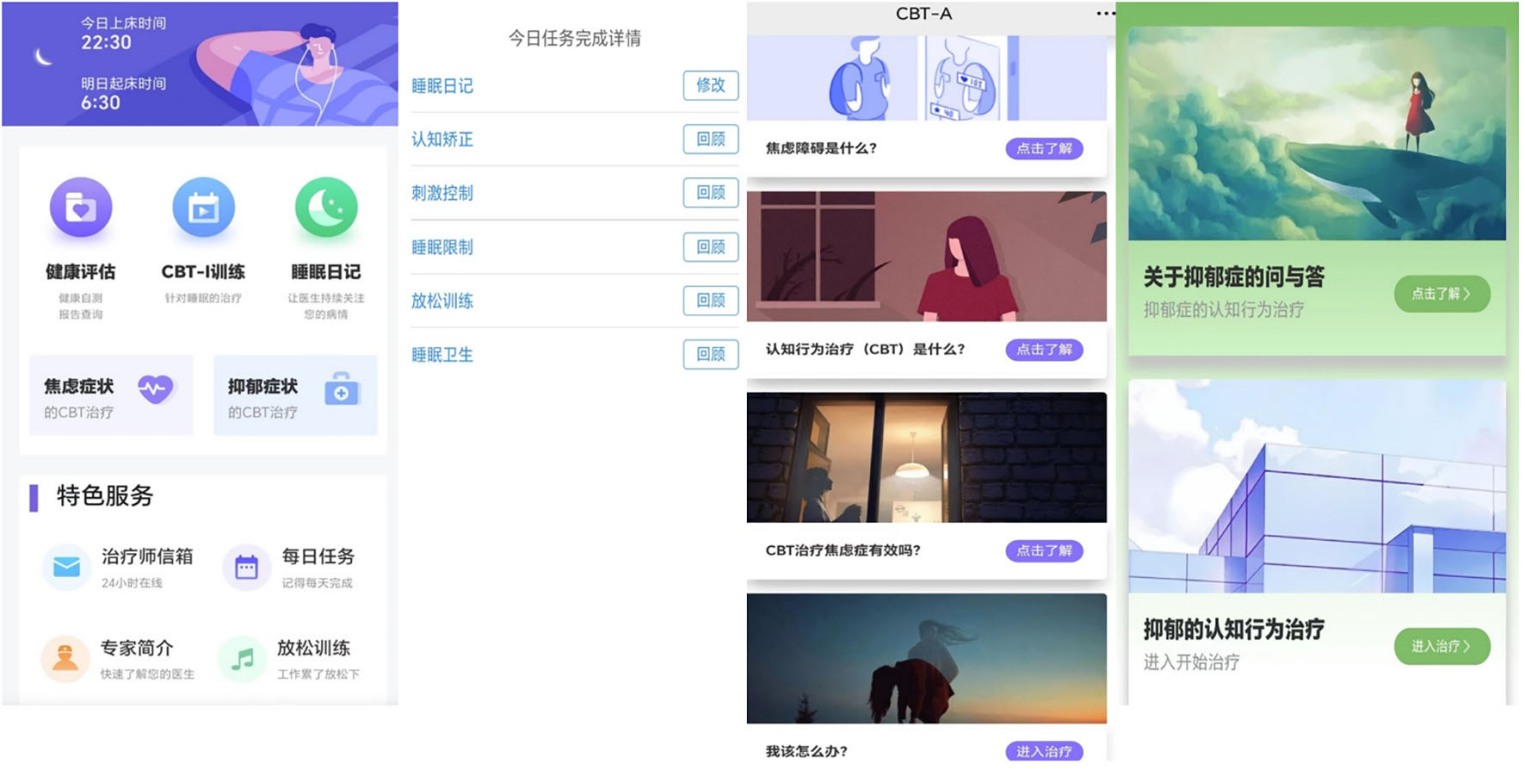
Screenshots of some representative interfaces (main interface, the task interface for sleep therapy, the anxiety and depression therapy interface).

The 5 insomnia treatment modules and 2 emotional intervention modules are as follows: Stimulus control is to enhance the connection between the bed and sleep and weaken the association between the bed and other activities such as eating, reading, etc., and thus making it easier for the patient to fall asleep. Restoring the bed to its function as a sleep-inducing signal. Sleep restriction is the process of improving sleep efficiency by reducing the amount of non-sleep time spent in bed. The main goal of cognitive therapy is to correct irrational cognitions related to sleep. Sleep Hygiene Education helps to establish good sleep habits. Relaxation training is used to regulate the patient’s emotional state. The treatment modules for anxiety and depression provide a wide range of self-help techniques for emotional intervention that are easy to follow and learn. In the anxiety module, it will help to identify the patient’s distorted cognition, conduct truthfulness testing, develop alternative thinking, etc. to correct the patient’s distorted cognition, and finally help the patient to accept themselves through positive self-suggestion. In the depression intervention session, we explained the knowledge about depression through voice and text, and then targeted intervention on depressive symptoms through mood logging, behavioral activation, task grading, and cognitive remediation. Screenshots of the homepage and part of the interface are shown in **Figure 2**.

In total, there are 7 specific therapy modules, intelligently arranged in a different order depending on each individual’s particular situation. Patients can review what they have learnt in the past and record their thoughts on the day. During the weekly sessions, the program automatically summaries the results of the treatment and gives feedback to the patients. For example, “Recently through your efforts, your sleep latency has been shortened by x minutes, your sleep time has been extended by x, and your sleep efficiency has increased by x percent”. This will give more confidence to the patients.

At the same time, we use the function of the therapist’s mailbox to achieve interaction with the patient, to supervise and help the patient to better understand and use the program, and to enhance and consolidate the effect of the treatment program. In addition, the program also set up a WeChat automatic reminder function, the daily pop-up “Hello, today’s task has not been completed”, when the patient is completed, the words of encouragement will appear “you have been adhering to the treatment for x days, congratulations, perseverance is the victory”.

### 2.6 Participant timeline

At baseline and at the end of the intervention, patients will be subjected to a series of psychological assessments, including sleep and mood symptoms. For patients in the study group, at the end of the 35-day intervention, a random sample of 30 patients will also be given a one-on-one qualitative interview to discuss their experience with Smart CBT-I plus, the interview results will be recorded. All patients will be evaluated at follow-up at 6 and 12 months after completion of treatment. Patients will be made aware of all assessment results. They will also be able to view their weekly assessments on their own in the program. they will still be able to use the program on their own after the 35-day session. The specific follow-up schedule is shown in **Table 1**.

**Table 1:** Subject visit schedule.

items	Screening visit	Baseline visit	Post-intervention visit	Follow-up visit	
Visit	V0	V1	V2	V3	V4
Time	Day -7-0 * (± 1 day)	Day 0 (± 1 day)	35 days after baseline (± 3 days)	6 months after intervention (± 7 days)	12 months after intervention (± 7 days)
Sign informed consent	**×**				
Inclusion/exclusion criteria	**×**	**×**			
Self-made general demographic questionnaire		**×**			
Self-made clinical feature collection scale		**×**			
Semi-structured sleep assessment questionnaire		**×**			
Insomnia Severity Index Scale (ISI)	**×**	**×**	**×**	**×**	**×**
Hamilton Depression Scale (HAMD)	**×**	**×**	**×**	**×**	**×**
Hamilton Anxiety Scale (HAMA)	**×**	**×**	**×**	**×**	**×**
Clinical Gross Impression Scale (CGI)		**×**	**×**	**×**	**×**
Epworth Sleepiness Scale (ESS)		**×**	**×**	**×**	**×**
Pittsburgh Sleep Quality Index Scale (PSQI)		**×**	**×**	**×**	**×**
Hygiene economics evaluation		**×**	**×**	**×**	**×**
Adverse event report form			**×**	**×**	**×**
Serious Adverse Event (SAE) report form			**×**	**×**	**×**
Six quality of life questionnaires		**×**	**×**	**×**	**×**
Generalized Anxiety Scale (GAD-7)		**×**	**×**	**×**	**×**
Dysfunctional Beliefs and Attitudes about Sleep Scale (DBAS-16)		**×**	**×**	**×**	**×**
Rapid Report of Depressive Symptoms - Self-report score 16 items (QIDS-SR16)		**×**	**×**	**×**	**×**
Life Event Scale (LES)		**×**			
Therapeutic Alliance Questionnaire Revision (WAI-S)			***×***		
Outline of semi-structured qualitative interviews (30 patients of study group)			***×***		
medication administration record	**×**	**×**	**×**	**×**	**×**

### 2.7 Sample size

The sample size required for the study was calculated using G*power software using the formula:


$$n_{C}=\frac{{\left(\begin{array}{c} Z_{1-\alpha }+Z_{1-\beta } \end{array}\right)}^{2}}{{\left(\begin{array}{c} \pi_{T}-\pi_{C}-\text{Δ} \end{array}\right)}^{2}}\left[\frac{\pi_{T}\left(\begin{array}{c} 1-\pi_{T} \end{array}\right)}{K}+\pi_{C}\left(\begin{array}{c} 1-\pi_{C} \end{array}\right)\right]$$

The p is equal to 0.05, the statistical test power is equal to 0.80. Previous studies have suggested that CBT-I usually has a medium potency, that is, an effect size of medium 0.5 (34). Referring to the relevant literature, the average shedding rate of CBT-I was about 15% (33, 37), obtaining the required minimum sample size of 60 cases for the psychoeducational group (control group) and 120 cases for Smart CBT-I plus (study group), for a total of 180 cases.

### 2.8 Discontinuation

Patients may choose to withdraw or be asked to withdraw from this study if the following conditions are met:

- Any time the patient wants to withdraw.
- Serious adverse event.
- Combined use of prohibited therapies (including Morita therapy, Vipassana therapy, systematic individual psychotherapy, group psychotherapy, MECT, rTMS, etc.).
- Serious physical illness.

Poor compliance(e. patients absent from treatment for 15 consecutive days or more).

Patients should be clearly informed of their right to withdraw from the study at any time, regardless of their stage.

The Principal Investigator is responsible for deciding whether to withdraw the patient from the study after consultation with the members of the research team.

## 3 Outcome measurements and instruments

This study will use both self-assessment and other-assessment scales, and the study assessments will be completed partly by the patients online in self-help and partly with the help of an assessor. Analyses of these scales will be conducted by a statistician without knowing how the patient groups will be assigned.

The efficacy evaluation indicators include (1) Main indicators:

Between-group comparison of ISI scores between the two groups after intervention and within-group comparisons before and after the intervention.

The ISI is a brief sleep self-assessment tool with 7 items to evaluate the severity of a patient’s insomnia (38). Using a single-item scale from 0 to 3, it is primarily used to evaluate the severity of insomnia in patients. The ISI is highly sensitive, valid, reliable, brief, easy to administer, statistically easy to use, provides relevant information for diagnosis and treatment, and is a reliable tool for quantifying the perceived severity of insomnia. The intervention will be considered successful when, after 35 days, a significant difference in the ISI scores between groups is found in the study group compared to the control group. Also, within-group comparisons between the two groups before and after the intervention will be explored thereby confirming the effect of the intervention more clearly.

(2) Secondary indicators:

a. Between-group comparison of the total HAMA score reduction rate in the two groups of patients;

b. Between-group comparison of the HAMD total score reduction rate in the two groups of patients;

The HAMA (39)and HAMD (40, 41) are scales that measure the severity of anxiety and depressive symptoms and are considered to be among the most widely used scales with good reliability and validity in the measurement of anxiety and depression. The HAMA contains 14 items and the HAMD contains 17 items, most of which are scored on a 4-point scale (from 0 to 4).

c. between-group comparison of sleep latency reduction time before and after intervention in the two groups of patients;

d. intergroup comparison of the time of increase in total sleep duration before and after intervention in the two groups of patients;

e. Comparison between the two groups of patients before and after the intervention of the Pittsburgh Sleep Quality Index (PSQI) total score reduction rate and the change of each factor score;

(3) Other psychological scales:

The PSQI is a standardized self-administered questionnaire developed by Buysse et al. (1989), is one of the most widely used standardized scales for assessing the subjective quality of sleep (42). It consists of 19 self-assessment and 5 other-assessment entries, each calculated on a scale of 0 to 3, with “0” referring to no difficulty, “1” referring to mild difficulty, “2” referring to moderate difficulty, and “3” referring to severe difficulty, and the cumulative scores for each factor are the total score of the PSQI, which ranges from 0 to 21, with the higher scores indicating poorer quality of sleep, and ≥8 suggesting the presence of sleep problems.

f. Between-group comparisons of improvements in sleep beliefs and attitudes (DBAS-16) before and after intervention in the two groups of patients;

Dysfunctional beliefs and attitudes about sleep (DBAS) (43) is primarily used for cognitive situations related to the evaluation of sleep and is a self-assessment tool for misconceptions about sleep. Morin et al. proposed a simplified 16-item (DBAS -16) version of the original 30-item scale in order to simplify its administration and increase the usefulness of the scale. Subjects rate the ideas in the scale on a visual scale. Eleven numbers from 0 to 10 are marked on a 100 mm long line. 0 means strongly disagree and 10 means strongly agree.

g.The number of remitters and responders of study group.

Applicability Indicators: a. All-cause shedding rate;

b. Patient experience based on semi-structured qualitative interviews. The interview outline centered on the patient’s experience of using Smart CBT-I plus treatment, such as: how do you feel about Smart CBT-I plus, how was the experience during the use, what impressed you or what was the most impressive content or fragment in the use, etc. This interview will be terminated when the patient has no new expression related to his experience;

c. Patient completion rates with Smart CBT-I plus;

d. Average usage time of the application.

## 4 Statistical analysis

The datasets included in the statistical analyses will be divided into the Full analysis set (FAS) and the Per Protocol Set (PPS). FAS refers to people who have had at least one follow-up after enrollment and have data on key indicators. Cases missing baseline data for the primary evaluation indicator will be excluded. PPS refers to cases that meet the inclusion and exclusion criteria on the basis of FAS, have valid baseline values, have good adherence, and do not violate the clinical study protocol. Data will be analyzed using both the FAS and the PPS, with FAS results being the mainstay.

Statistical tests will be performed using SPSS version 23.0 software and relevant data will be considered statistically significant if P < 0.05. Comparisons between groups will be made using chi-square analysis, continuous variables will be statistically described using mean ± standard deviation (M ± SD), comparisons between the two groups will be made using the independent samples t-test, and repeated measures such as ANOVA will also be used to compare the degree of symptomatic improvement in the intervention group and the control group and to analyze the group × time interaction. In addition, Binary Logistics regression and COX regression will be used to compare the changes in each indicator before and after the intervention between the two groups. Multiple imputation will be used for handling missing data.

Data will be analyzed using content analysis to extract patients’ experiences of using the Smart CBT-I plus. The audio recordings of the semi-sectional constructed interviews will be transcribed into text before the interview data is independently coded and analyzed by each of the 2 researchers, and if inconsistencies are encountered, they will be discussed to reach consistent conclusions, and inter-researcher coding consistency will be used to rate the study’s reliability.

## 5 Discussion

This online trial will test the effectiveness in terms of clinical outcomes and applicability of a self-help intervention platform to help alleviate insomnia combined with anxiety and/or depressive symptoms, a common clinical condition.

There are several limitations inherent in this study. Firstly, there is a possibility of dropout in either of the two groups. We will analyze and investigate the reasons for dropouts. Secondly, in terms of the applicability of this platform, older adults and patients with poor cognitive learning may have difficulty with self-help interventions, which in turn affects the validity and general applicability of this platform.

Thirdly, this self-help treatment relying on electronic technology may be more appealing to younger patients, who are more concerned with efficiency, time saving and personal privacy, and therefore prefer online interventions. Smart CBT-I plus lacks generalizability to different age groups.

Fourth, Smart CBT-I plus may be more suitable for patients with higher adherence and more positive psychological engagement, requiring more patient initiative, but it is not appealing to some patients with sleep disorders who have severe attention and energy deficits due to poor sleep and mood and who do not have sufficient motivation and conditions for active help-seeking.

This study also has several strengths. People with insomnia combined with depression and/or anxiety are a large group in China. In contrast, psychological resources and capacity to provide psychological interventions for this group are limited due to the shortage of qualified psychotherapeutic resources and the high cost of treatment. Therefore, it is particularly important to look for optimization techniques that use standardized procedures and produce positive results. Smart CBT-I plus can save costs such as time and economy, is more convenient and efficient, and has higher accessibility and applicability, which is the potential effects it has. Unlike previous mental health indicators involving the diagnosis of mental disorders such as insomnia, depression, anxiety, etc., this study chose symptoms rather than diagnostic criteria as inclusion criteria. Shifting from a disease-orientated intervention model to a health-orientated intervention model for accurate psychological self-help health interventions. These technologies have the potential to be further developed and made available to a wider population. At the same time, in the control group of this study, some popular science articles will be used to provide a mild intervention to the patients, and it will be verified what effect this will have and whether it has some value and significance.As a resource for non-stigmatizing and complementary psychological interventions that address, prevent, treat and promote mental health, these techniques are expected to have a sustainable positive impact on people’s mental healthcare and society at large.
