# Supplementary material for: The applicability and effectiveness of the cognitive behavioral therapy for insomnia (Smart CBT-I plus) online program in patients with insomnia disorder combined with anxiety and depression: a randomized controlled trial protocol
Source: Front Psychiatry. 2025 Mar 28;16:1450275. doi: 10.3389/fpsyt.2025.1450275 (PMC11986715; doi:10.3389/fpsyt.2025.1450275)
Supplement: Supplementary file 1 [file Table1.docx]

**Supplementary Table 1.**

The clinical trial protocol checklist

| **Section/item** | **ItemNo** | **Description** |
| --- | --- | --- |
| Administrative information | | |
| Title | 1 | The applicability and effectiveness of the Cognitive Behavioral Therapy for Insomnia (Smart CBT-I plus) online program in patients with insomnia disorder combined with anxiety and depression: a prospective randomized controlled trial protocol |
| Trial registration | 2 | The registration number of the Chinese Clinical Trial Registry is ChiCTR2400094145 |
| Protocol version | 3 | 17 Jun 2024，version1.1 |
| Funding | 4 | This project is funded by the "Science and Technology Innovation Action Plan" of Shanghai（Project number: 20Y11906600），and the hospital-level research project (Project number: 2021-YJ08) of Shanghai Mental Health Centre |
| Roles and responsibilities | 5 | For details, see the “Author contributions” section of the text |
| **Introduction** |  |  |
| Background and rationale | 6 | See “1.1 Background” section in the text for details |
| Objectives | 7 | See “1.2. Objectives” section in the text for details |
| Trial design | 8 | See “2.1. Trial design” section in the text for details |
| **Methods: Participants, interventions, and outcomes** | | |
|  |  |  |
| Study setting | 9 | See “2.2. Setting and recruitment” section in the text for details |
| Eligibility criteria | 10 | See “2.3. Participants and eligibility” section in the text for details |
| Interventions | 11 | See “2.5. Intervention＆2.8 Discontinuation” section in the text for details |
| Outcomes | 12 | See “3. Outcome measurements and instruments” section in the text for details |
| Participant timeline | 13 | See “2.6. Participant timeline Table 1 Subject visit schedule” section in the text for details |
| Sample size | 14 | See “2.7. Sample size” section in the text for details |
| Recruitment | 15 | See “2.2. Setting and recruitment” section in the text for details |
| Methods: Assignment of interventions (for controlled trials) | | |
| Allocation: | 16 | See “2.4. Randomization＆2.5. Intervention” section in the text for details |
| Methods: Data collection, management, and analysis | | |
| Data collection methods | 17 | See “2.6. Participant timeline＆3. Outcome measurements and instruments” section in the text for details |
| Data management＆Statistical methods | 18 | See “4. Statistical analysis” section in the text for details |
| Methods: Monitoring | | |
| Data monitoring | 19 | Data Collection will be completed through the Electronic Data Collection System of Shanghai Mental Health Center, which will be completely retained in the system |
|  |  |  |
| Harms | 20 | It will be clearly recorded in the Electronic Data Collection System of Shanghai Mental Health Center |
| **Ethics and dissemination** | | |
| Research ethics approval | 21 | This study has been approved by the Ethics Committee of Shanghai Mental Health Center |
| Protocol amendments | 22 | The researchers have completed the revision |
| Consent or assent | 23 | The evaluator will ask the enrolled subjects to sign the informed consent form |
| Confidentiality | 24 | The personal information of the relevant personnel will be entered into the electronic system, and only the leading researchers will have access to it |
| Declaration of interests | 25 | This study will be conducted in the absence of any business or financial relationship that could be seen as a potential conflict of interest |
| Access to data | 26 | Only the principal researchers have access to the final data set |
| Ancillary and post-trial care | 27 | The subjects in the control group will be free to use the Smart CBTI-plus after the end of the experiment, and this study is harmless to the subjects |
| Dissemination policy | 28 | The researchers will publish the results of the study in a scientific paper after the trial |
| **Appendices** |  |  |
| Informed consent materials | 29 | A model informed consent form is available |
